# Reactive shepherding along a dynamic path

**DOI:** 10.1038/s41598-024-65894-5

**Published:** 2024-06-28

**Authors:** Stef Van Havermaet, Yara Khaluf, Pieter Simoens

**Affiliations:** 1 IDLab, Department of Information Technology, Ghent University - imec, B-9052 Gent, Belgium; 2 Department of Social Sciences, Wageningen University and Research, 6706KN Wageningen, The Netherlands

**Keywords:** Shepherding, Reactive path planning, Computer science, Computational science

## Abstract

Shepherding, the task of guiding a herd of autonomous individuals in a desired direction, is an essential skill employed in the herding of animals, crowd control, and evacuation operations. Integrating shepherding capabilities into robots holds promise to perform such tasks with increased efficiency and reduced labor costs. To date, robotic shepherds have only been designed to steer a herd towards a predetermined goal location without constraints on the trajectory. However, the tasks of a sheepdog encompass not only steering the herd but also (i) maintaining the herd within a designated area and (ii) averting dangers, obstacles, or undesirable terrain such as newly sown land. We present a decentralized control algorithm for multi-robot shepherding designed to guide a group of animals along a specified path delineated by two boundaries. The algorithm incorporates the additional objective of preserving the group within these boundaries. Simulation results reveal that, especially in sections of the path with sharp turns and a small distance between the boundaries, the group exhibits a tendency to deviate beyond the prescribed margin. Additionally, our findings emphasize the algorithm’s sensitivity to the ratio of robot-group sizes and the magnitude of the group’s velocity.

## Introduction

In our rapidly changing environment, both anthropogenic and natural threats are placing immense pressure on animal populations, with fish being a poignant example. Illegal fishing^1^, habitat destruction^2^, and pollution^3^ are just a few examples of contemporary challenges. Using robots to guide and protect these vulnerable populations emerges as promising avenue for intervention. Robots could be used to assist in guiding fish away from hazardous zones^4^, directing them along secure trajectories, and monitoring environmental parameters to ensure the well-being of the population^5^.

The term shepherding was originally coined to describe the process by which dogs guide and direct sheep towards predetermined destinations. The field of robotics has adopted this terminology to characterize tasks involving robotic agents herding various animals, including sheep^6–8^, cattle^9^, and ducks^10^, enabling crowd control^11^, averting bird-aircraft collisions^12,13^, or safely evacuating people^14^. Robotic agents, designed with distinctive visual cues or equipped with actuators eliciting aversive responses from animal groups, can employ analogous shepherding mechanisms as naturally perceived threats, akin to dogs herding sheep^15^. The collective behavior displayed by these shepherded animal groups encompasses both aggregation and evasion dynamics in response to potential threats^16^.

In this study, we are moving beyond the conventional shepherding task of guiding animals towards predetermined destinations. Our algorithm is designed to guide an animal collective along a secure path, as opposed to merely steering them toward a fixed goal location. The path is demarcated by two boundary lines. This area is constructed by enveloping a series of line segments with a margin. The margin width varies as the path unfolds, simulating more realistic scenarios where the safe zone exhibits variability. We assume that the herded individuals lack the capacity to discern the pathway autonomously, indicating that their motion remains unaffected by the state and structure of the path. This assumption reflects real-world applications where the environment may harbor hidden dangers for the group that only become apparent after damage has occurred to the animals, such as in the case of an oil leak. Alternatively, the area might need to be avoided for ecological or economic reasons, such as preventing the group from grazing on newly seeded fields.

The Robot Sheepdog Project, led by Vaughan et al.^17^, was a pioneering initiative in the realm of shepherding problem solutions. They devised a novel approach using force vectors inspired by Reynolds’ inter-individual rules for flock behavior^18^. The single robot strategically positioned itself on the opposing side of the flock with respect to the goal and moved towards them. However, it is crucial to note that the project’s shepherding method had a limitation, as it confined the ducks to move along the periphery of an enclosed environment until reaching the goal. Some follow-up works have built upon the aforementioned algorithm to extend its applicability to unconstrained environments, where individuals naturally disperse^19–21^. In the work by Miki and Nakamura^22^, a shepherd dynamically alternates between guiding the cohesive group and gathering dispersed individuals. As a consequence, the shepherding agent displays the behavior of real sheepdogs, moving side-to-side behind the group. Their experiments further demonstrated that employing two shepherds is more efficient in guiding the group than having only one. This aligns with other studies suggesting that single-agent solutions are constrained in handling larger group sizes^23^. Therefore, multiple shepherds prove to be more effective in controlling larger groups^24^. However, the majority of shepherding control approaches presume that the shepherds possess global knowledge of the positions of every individual in the environment^25,26^. Addressing the challenge of guiding a group to a goal with multiple shepherds, utilizing only local information, was first tackled by Lee and Kim^27^. In their approach, shepherds coordinate to aggregate one cohesive group. Some shepherds steer wandering members towards the main group, while others concentrate on maintaining the position of the main group.

Similar to Lee and Kim, the algorithm we propose operates within a distributed fashion, without any central control unit. Our work extends beyond theirs as our robots utilize local observations to collectively guide a group along a safe path, as opposed to guiding it toward a goal location without trajectory constraints. Decentralized multi-robot systems offer advantages over centralized ones by eliminating a single point of failure. Moreover, our algorithm is designed to operate without prior knowledge of the environment. Each robot determines its next action based on (i) the positioning of other robots and fish in proximity, and (ii) locally observed pathway information. This approach ensures that the entire group is guided towards the goal location while remaining within the safe path. The proposed algorithm is an extension of our previous work on cage formation^4^, and now simultaneously employs a strategy to maintain a cage around the group and nudge the group in the desired direction along the path.

Steering a group while preserving a caging formation was first tackled by Varava et al.^28^. Their proposed solution relies on a centralized RRT-based (rapidly-exploring random tree) algorithm, incorporating computational topology techniques to validate caging formations. However, their approach assumes access to global information and starts from an already formed caged formation. Our approach is inspired by the behavioral rules of wolf-pack hunting strategies^29^, where the hunting wolves strategically position themselves to evenly spread out across the contour of the group. To replicate this behavior, our robots self-organize through the implementation of two simple decentralized rules:: (i) a robot moves toward the group until a specified distance threshold is reached, and (ii) when in close proximity to the group, the robot moves away from other nearby robots. By implementing these results, the robots effectively construct a cage around the animal group. The cage serves the purpose of preventing any potential escapes, and additionally, this strategy allows the robots to gather new information about the surrounding area before the group interacts with it. This information can be crucial for the timely redirection of the group away from undesirable areas. Once the cage has been formed, the robots strategically nudge the group in the desired direction along the path while maintaining the caging formation. Drawing from previous shepherding research, a fish is modeled to move in the opposite direction of a robot when the relative distance is below a certain threshold. Consequently, each robot decides between approaching to push the fish or staying further away to create free space for the fish to move freely. While guiding the group, robots might temporarily move beyond the boundaries of the path, but it is essential for the group to always remain within these boundaries. This temporary deviation can occur due to various reasons, including delays in the robots’ response caused by variations in the speed at which they detect the boundaries and initiate maneuvers. Additionally, it can result from encountering narrow or sharp turns in the path.

We investigate the impact of various parameters on the efficacy of our shepherding algorithm. These parameters encompass the characteristics of the path (i.e. the space between the boundaries and the degree of a turn), the size of the system which consists of the number of robots and fish, and the maximum velocities of the robots and fish. Additionally, we investigate the correlation between the success of the caging phase and the overall success of the shepherding process. This multifaceted examination aims to provide insights into the complex dynamics at play and to shed light on the algorithm’s performance under different conditions and configurations. We found that the success of the caging is dependent on the magnitude of the fish’s maximum velocity. Additionally, we observe that the success of the shepherding process may decline when navigating sharp turns along the path, especially in regions where the spacing between the boundaries narrows. This decline is contingent upon the system size, as well as the space between the boundaries.

Our findings offer valuable insights that can inform the analysis and refinement of complex shepherding algorithms, especially in scenarios involving hazardous and undesirable areas, across a spectrum of applications. These include biological^30^, environmental^31^, and robotics applications^25^. Furthermore, our research may find utility in the study of animal and insect collectives, where one species may need to guide, hunt, or gather individuals of another, providing a bridge between the natural systems and innovative technological solutions.

## Results and discussion

**Figure 1:**
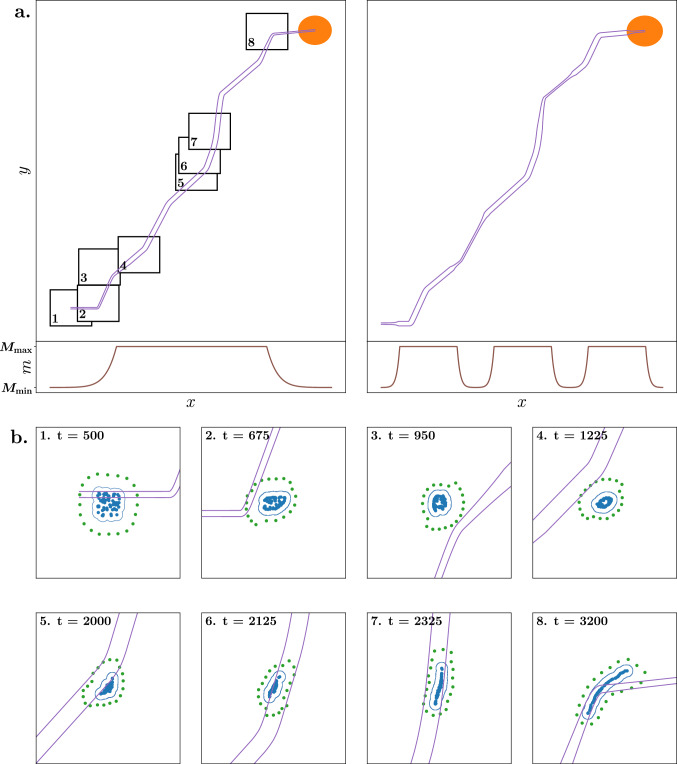
**(a)** Visualization of two different paths (first row), based on the same sequence of linesegments. The respective margin width of each path is shown below (second row). The margin of the left path is generated based on a single Gaussian, while the right margin is generated based on a 3-component Gaussian mixture. The goal location is illustrated by a full orange circle. **(b)** Visualization of the robots (green dots) shepherding the herd (blue dots) along a path with varying margins (purple lines) at different time steps. The location of the time step figures in (**a**) are respectively shown on the left path. The contour of the herd is marked by a blue line. During the initial time steps ($t < 500$), the herd is positioned at the start of the path and the robots construct the caging formation. Then, the robots try to move the herd inside the margin along the local direction of the path. After some time, the robots are able to maintain the herd inside the margin, until a steep turn occurs at a narrow point of the path ($t = 3200$). Generated using $N_R = 20$, $N_G = 60$, and $M_{\min } = 10$.

In the experiments, $N_R$ robots are tasked with guiding an animal group of size $N_G$ along a safe path, which is defined as the area between two boundary lines. Starting from a series of line segments that evolve monotonically along positive *X* and *Y* direction, the path boundaries are constructed by applying an orthogonal margin at both sides of the line segments. This margin width is sampled from a truncated mixture of *K* Gaussian distribution components, with the minimum margin width $M_{\min }$ as a parameter and the maximum derived as $M_{\max } = 3 M_{\min }$ (see Methods). An example of a randomly generated path is shown in Fig. 1a for two different Gaussian mixtures (left $K=1$, and right $K=3$).

The purpose of the shepherding algorithm is to advance the animal group along the path while ensuring the group remains within the boundaries of the safe path. Figure 1b depicts an experimental instance wherein the robots strive to cage and shepherd the group inside the boundaries at different points in time. When a robot enters a defined radius around an animal, the animal responds by moving away from the robot in the opposite direction. Simultaneously, the animal group exhibits collective behavior, aligning their orientation and maintaining cohesion. Through the utilization of these interaction behaviors, the robots adeptly manipulate the movement of the group. The performance of the shepherding algorithm is defined as the percentage of group animals that is within the safe path, denoted by $\rho$. The performance of caging is measured by the percentage of the group that is not inside the cage, denoted by $\psi$. In case the robots fail to construct a closed cage formation, we set $\psi = 100$, i.e. the whole group is outside the cage. The performances $\rho$ and $\psi$ are both measured at any x-coordinate of the path.

We conducted simulation experiments to assess the impact of the considered control parameters on the shepherding and caging performances of the proposed algorithm. The control parameters are different minimum margin intervals of $[M_{\min }, 3 M_{\min }]$ with $M_{\min }\in \{10, 20, 50\}$, system sizes which consist of the number of robots and animals $(N_R, N_G) \in \{(20, 30), (20, 60), (40, 60)\}$, and animal maximum velocities $v_G \in \{2, 6, 10\}$ with robot maximum velocities $v_R \in \{\frac{3}{2} v_G, 2 v_G, 3 v_G\}$. Each experiment with the same configuration is iterated 30 times, each time employing different seeds. A single simulation run ends when either the time limit of 5000 time steps has been surpassed or the group has reached the goal location at the end of the path. In what follows, we study the relationship between the performance and (i) the challenging characteristics of the path, (ii) the system size, and (iii) the robot/animal velocities.

### Shepherding performance declines at path narrowing and sharp turns

**Figure 2:**
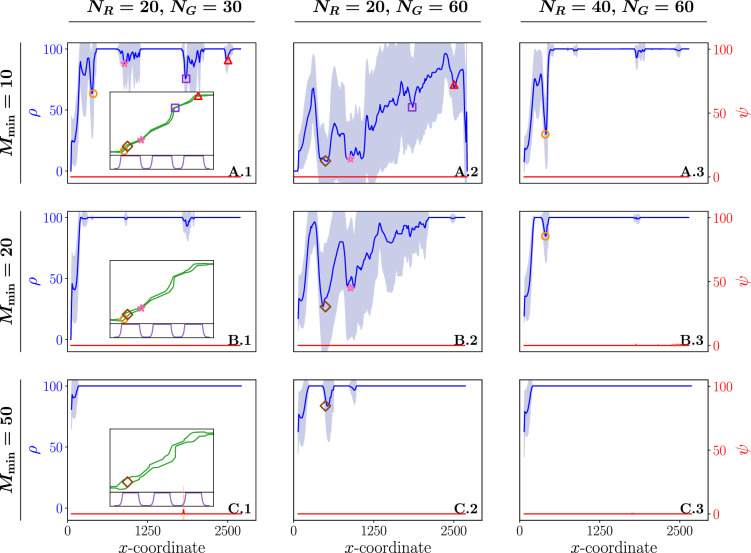
The percentage of the group within the safe path (denoted as $\rho$, colored in blue) and the percentage of the group that is not caged (denoted as $\psi$, colored in red) in function of the mean *x*-coordinate of the group. Each row corresponds to simulation results for different values of the minimum margin width $M_{\min }$ of the path: 10 (**A.1-3**), 20 (**B.1-3**), 50 (**C.1-3**). Each column considers different sizes of the system (i.e. the number of robots $N_R$ and the size of the group $N_G$): $N_R = 20, N_G = 30$ (**A-C.1**), $N_R = 20, N_G = 60$ (**A-C.2**), $N_R = 40, N_G = 60$ (**A-C.3**). The first column shows the corresponding path of its row as an inset. Significant declines in performance are marked by different colors and symbols: a brown diamond, an orange circle, a red triangle, a purple square and a pink star. The location on the path where these declines happen is correspondingly marked in the insets. The mean (solid line) and variance (shaded area) of $\rho$ and $\psi$ results from averaging over 30 stochastically independent simulations for each configuration, with $K = 3$.

Figure 2 displays the percentage of the group within the safe path ($\rho$) and the percentage of the group not caged ($\psi$), as a function of the mean *x*-coordinate of the group. Each column showcases outcomes for distinct system sizes, while each row varies in the minimum margin width $M_{\min }$ (A: 10, B: 20, C: 50) used to generate the margins. All paths were constructed from the same series of line segments but differ in margin widths on both sides of these segments. A visual representation of each path is provided as an inset in the first column of the corresponding row (A-C.1).

For paths with the smallest considered margin width ($M_{\min }= 10$), varying patterns emerge based on the different system sizes. In the instance of a system with a 20/30 robot-animal composition (A.1), optimal shepherding performance ($\rho = 100$) is achieved; however, this is intermittently interrupted by several decline points in $\rho$. Observing these points on the path, we note that declines tend to occur where the margin width is minimal or a sharp turn occurs. Despite the sudden decline in $\rho$ at these points, the group remains fully caged ($\psi = 0$), allowing these systems to recover and eventually reach optimal performance again. Increasing the number of animals to a system of size 20/60 (A.2), introduces notable $\rho$ variance for every *x*-coordinate, with mean $\rho$ never reaching optimum. Notably, this system is the only one among the considered systems where not all animals reach the desired goal, as shown in Supplementary Fig. S1.1a. This observation is also reflected in Fig. 2A.2, where $\rho$ is not optimal at the maximum x-coordinate. Once again, substantial declines in shepherding performance can be detected, occurring mostly (all except the one indicated by an open brown diamond) at the same points in the path as observed for the 20/30 system size. Doubling the number of robots to a system of 40/60 robot-animals (A.3), the system becomes impervious to the narrow areas in the middle of the path. The system attains a stable optimal state, wherein it consistently maintains its maximum performance ($\rho = 100$ and $\psi = 0$) over a substantial distance along the path. Prior to reaching this stable optimal state, the system appears to necessitate some initial distance to stabilize, notably interrupted in the first turn of the path, as indicated by the open orange circle. Comparing 20/30 (A.1) and 40/60 (A.3) systems with a constant 2/3 ratio reveals distinct performances. The former consistently faces disruptions, while the latter achieves a stable optimal state. This suggests a non-linear relationship between system size and $\rho$, which we investigate in the following subsection.

Across all system sizes considered, shepherding performance improves when increasing the minimum margin width to $M_{\min }= 20$, resulting in a margin in the interval [20, 60]. Specifically, declines in $\rho$ disappear for the 20/30 system size (B.1) and the system reaches a stable optimal state. For a system of size 20/60 (B.2), the number of declines decreases, and the system eventually reaches optimal performance at the end of the path. Although the decline indicated by an open orange circle persists in the system of size 40/60 (B.3), it is substantially weaker compared to the one observed for $M_{\min }= 10$ (A.3). Further increasing $M_{\min }$ to 50, all considered system sizes achieve a stable optimal state that persists until the end of the path after an initial stabilization period. Supplementary Fig. S2.1 shows that for paths with a longer narrow segment at the start of the path (by generating margins with $K=1$), in comparison to the paths of Fig. 2 ($K = 3$), the systems exhibit a significantly later stabilization point. The delay is attributed to the robots facing challenges in aligning the group within the path at the beginning, causing a delay in achieving the desired level of stability.

**Figure 3:**
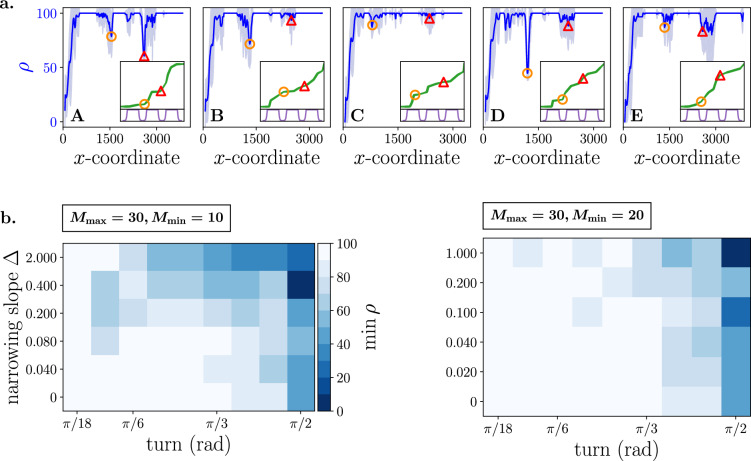
**(a)** The percentage of the group within the safe path ($\rho$) as a function of the mean *x*-coordinate of the group across five randomly generated paths. Each path is individually displayed as an inset alongside the corresponding results, featuring a visualization of the margin. Notable declines of $\rho$ are denoted by distinct colors and symbols, specifically an open red triangle and an open orange circle. These dips are located along the path and are similarly marked in the insets. The mean (solid line) and variance (shaded area) of $\rho$ results from averaging over 30 stochastically independent simulations for a configuration with $N_R=20$, $N_G=30$, $M_{\min }=10$ and $K = 3$. **(b)** The minimum percentage of the group inside the safe path ($\min \rho$), measured within the local range of the turn. Each path consists of only one turn, where the margin width decreases linearly from $M_{\max } = 30$ to $M_{\min }$ with a (narrowing) slope $\Delta$. The left and right panels respectively show the results for $M_{\min } = 10$ and $M_{\min } = 20$. The values of $\min \rho$ represent the mean over 5 seeds for every parameter configuration of the turn, minimum margin width, and narrowing slope. Other parameters $N_R = 20$ and $N_G = 30$ are constant.

To explore the impact of path characteristics on declines in $\rho$, we conducted experiments with 20 robots and 30 animals on five diverse paths, as depicted in Fig. 3a. These paths feature distinct series of line segments but share the same margins, generated with $M_{\min }= 10$ and $K = 3$. The selection of $K= 3$ allows us to observe multiple instances of narrowing within a single path, while variations in the line segments introduce different degrees of turns. Notably, when margin narrowing occurs along a straight path segment (e.g., open red triangles in B, C, D, and E), the performance decline is relatively small. Similarly, a sharp turn in a wider segment of the margin leads to a minor decline (e.g., the open orange circle in C). The most significant declines occur when a sharp turn happens within a narrow path segment (e.g., the open red triangle in A). Moreover, it appears that the greater the turn, the more pronounced the decline (as observed in the comparison of the open orange circles between B and D). To more precisely investigate what constitutes a *sharp* turn and a *narrow* segment, we conducted additional experiments with $N_R = 20$ and $N_G = 30$, involving paths consisting of only one turn. In these experiments, the margin width decreases linearly from $M_{\max } = 30$ to $M_{\min }$ with a (narrowing) slope $\Delta$. In Fig. 3b, the minimum percentage of the group inside the path ($\min \rho$), within the local range of the turn, is depicted for every parameter configuration. A turn of $\frac{\pi }{2}$ radians is evident to cause a substantial decline in $\rho$ for any narrowing slope, even when the margin width does not decrease ($\Delta = 0$). Notably, when the margin reduces three times in width ($M_{\max } = 30, M_{\min }= 10$), performance experiences a more significant decline with a faster reduction in margin width (i.e., an increase in the slope $\Delta$) and a larger turn. Conversely, when the margin only reduces 1.5 times in width ($M_{\max } = 30, M_{\min }= 20$), performance is more influenced by the turn than the narrowing slope.

### The system size plays a crucial role in reaching and maintaining a stable optimal state

**Figure 4:**
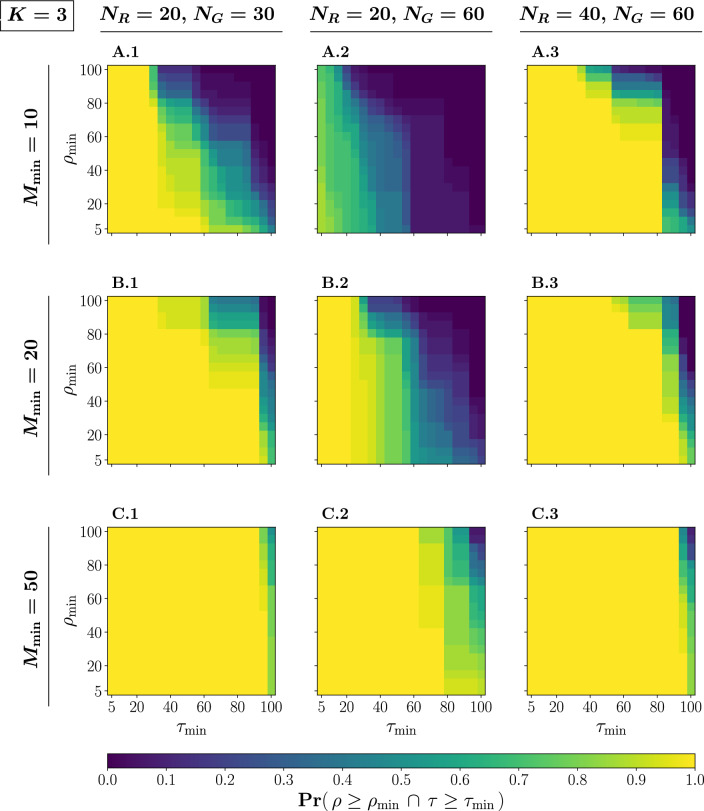
Probabilities $\Pr (\rho \ge \rho _{\min } \cap \tau \ge \tau _{\min })$ of the system maintaining at least a specified percentage of the group inside the safe path ($\rho _{\min }$) for at least a specified percentage of the total path length, measured on the *x*-axis, continuously ($\tau _{\min }$). Each row corresponds to simulation results for different values of the minimum margin width $M_{\min }$ of the path: 10 (**A.1-3**), 20 (**B.1-3**), 50 (**C.1-3**). Each column considers different sizes of the system (i.e. the number of robots $N_R$ and the size of the group $N_G$): $N_R = 20, N_G = 30$ (**A-C.1**), $N_R = 20, N_G = 60$ (**A-C.2**), $N_R = 40, N_G = 60$ (**A-C.3**). Probabilities are obtained by averaging over 30 stochastically independent simulations with $K=3$.

To explore in more detail the relationship between the size of the system ($N_R, N_G$) and the percentage of the group inside the safe path ($\rho$), we define $\tau$ as the continuous percentage of the total path length measured on the *x*-axis. Figure 4 presents the probabilities $\Pr (\rho \ge \rho _{\min } \cap \tau \ge \tau _{\min })$ across various values of $\rho _{\min } \in [5, 10,..., 100]$ and $\tau _{\min } \in [5, 10,..., 100]$, considering the same configurations of system sizes and minimum margin widths as previously explored.

We observe that there is no assurance that the robots can maintain the entire group within the safe path for the entire path length, as $\Pr (\rho = 100 \cap \tau = 100) < 1$ for each configuration. Interestingly, only configuration (C.1) exhibits a non-zero probability. This observation aligns with the findings in Fig. 2, where, even with the largest margin widths ($M_{\min }= 50$), the system requires some initial distance to reach a stable optimal state and thus $\rho < 100$ at the beginning of the path. We identify a dual factor contributing to this initial stabilization requirement. Firstly, the group must adapt its initial shape to fit the margin width. As illustrated in Fig. 1b, at $t=500$ (1), the contour around the group exhibits a relatively large circular shape. To ensure the entire group remains inside the safe path, the robots must subsequently coerce the group into a more rectangular shape, achieving this feat at time step $t = 2325$ (7). Secondly, the robots are required to guide the group to align with the current direction of the path. When a part of the group is influenced to alter its direction, this information must propagate across the entire group, a process that does not occur instantaneously. Figure 4 demonstrates that the majority of configurations complete the stabilization process since they maintain a stable optimal state for most of the path. However, systems with 20 robots and 60 animals encounter challenges in achieving a stable state, particularly for minimum margin widths of 10 (A.2) and 20 (B.2). Even though successfully caging 60 animals with only 20 robots is achievable, the system faces difficulties in consistently keeping the entire group within the safe path.

**Figure 5:**
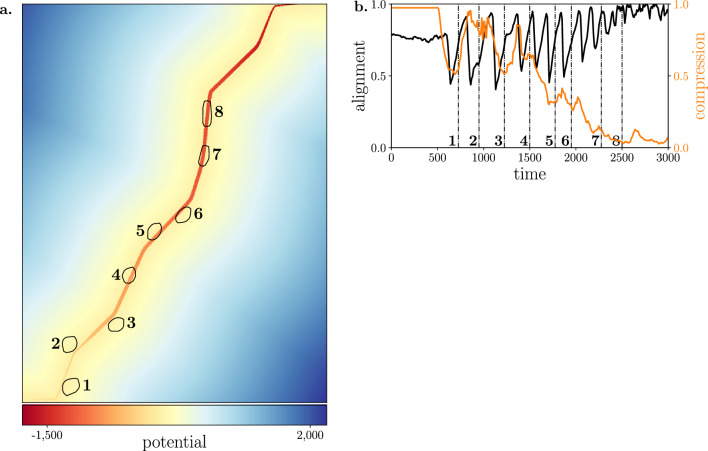
**(a)** The positions of the robots and the group are drawn by the convex hull of all individuals (depicted by the black contour) at different time points (chronologically labeled). The movement illustrates the crossing behavior, where the group sequentially transitions from one side of the path to the other. The colormap illustrates the artificial potential field of the environment, with the global minimum located at the end of the path. Outside the path, the potential diminishes toward the boundaries. Within the path boundaries, the potential decreases along the *x*-axis. These positions were extracted from an experiment with configuration parameters $N_R = 20$, $N_G = 60$, $M_{\min } = 10$ and $K = 1$. **(b)** The group-path alignment (black) as the average alignment between each animal’s direction and the desired direction along or towards the path, with a measurement of 1 indicating full alignment. The group compression (orange) measures how compact the spatial arrangement is. A circular formation corresponds to compression equal to 1, while a stretched-out group (adhering to the path geometry) results in compression as 0. The analysis was conducted on the same experiment as presented in (a). Both metrics are plotted over time, with different time points (annotated at the corresponding vertical lines) aligning with those in (**a**).

When we further observe the behavior of these systems, we find this to be a consequence of the limited time available to the robots for executing precise maneuvers required to redirect the large group of 60 animals. The detection of the path orientation change is a local process, and by the time the robots react, the narrow margin of 10 or 20 has already been breached. Consequently, a sequence of transitions from one side of the path to the other occurs, which we also refer to as crossing behavior. Figure 5a visually depicts this behavior, highlighting the intricate interplay of the aforementioned dual factors. Over time, as the spatial arrangement of the group gradually conforms to the shape of the path, the robots can more rapidly realign the group towards the path, eventually achieving stability within the path boundaries. We systematically explore nuanced aspects through a more detailed analysis (see Methods for the mathematical definitions of the following metrics). Group-path alignment is quantified as the average alignment between each animal’s direction and the desired direction along or towards the path, with a measurement of 1 indicating full alignment. Additionally, we measure group compression to assess how compact the spatial arrangement is. A circular formation corresponds to compression equal to 1, while a stretched-out group (adhering to the path geometry) results in compression as 0. Figure 5b illustrates how the group undergoes rapid oscillations in aligning with the current path direction. The severity of these oscillations gradually mitigates over time, until complete alignment with the path is achieved. Simultaneously, as the alignment process undergoes damping, the spatial arrangement of the group eventually conforms to the shape of the path, leading to a decrease in compression.

While Fig. 5 illustrates an experiment instance where the system of (B.2) eventually attains a stable state after the crossing behavior, this outcome is not consistent across all instances. Moreover, the duration of the crossing behavior varies inconsistently among systems with configurations of (A.2) or (B.2). If we revisit Fig. 4B.2, it becomes apparent that for a given value of $\tau _{\min }$ (e.g., 50), the probability diminishes as $\rho _{\min }$ increases but consistently remains above zero. This suggests that, in some experiments, the entire group remains within the safe path, while in others, only 20% of the group remains within the path for half the length of the path. This phenomenon accounts for the high variance observed in configurations (A.2) and (B.2) in Fig. 2. In comparing configurations (A-B.2) with (A-B.3), it is evident that deploying additional robots enhances the reliability of reaching and maintaining a stable state. However, upon comparing the outcomes between the 20/30 and 40/60 systems, the relationship between the system size and the duration of the stable state does not appear linear.

**Figure 6:**
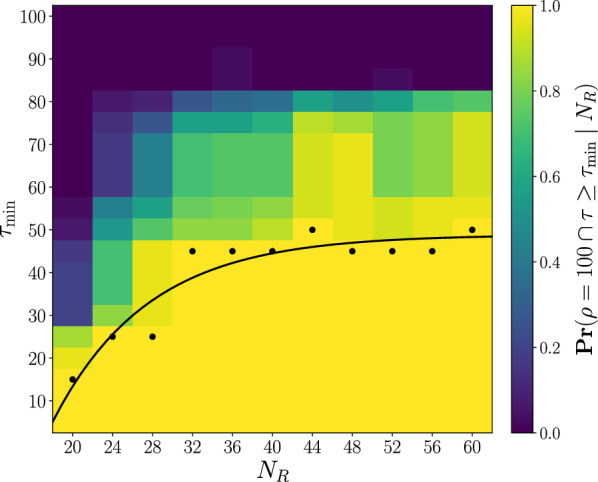
Probabilities $\Pr (\rho = 100 \cap \tau \ge \tau _{\min } \mid N_R)$ of the system maintaining the entire group inside the safe path ($\rho = 100$) for a percentage of the total path length measured on the *x*-axis ($\tau _{\min }$) in function of the number of robots ($N_R$). Solid black line serves as a guide to the eye, where the probability is greater than 0.975 for the highest value of $\tau _{\min }$, i.e. $\tau _{\min }'$. The curve results from non-linear regression to the exponential function $\tau _{\min }' = a \exp (-b \cdot N_R) + c$ with $a = -289.5, b = 0.1050$, $c = 48.86$ (residual standard error is 4.488). Probabilities are obtained by averaging over 30 stochastically independent simulations for each value of $N_R$, with $N_G = 60$, $M_{\min } = 20$, $K = 3$.

Figure 6 offers a comprehensive analysis of the influence of deploying additional robots on the effectiveness of the proposed algorithm. Specifically, it presents the probabilities of a system with $N_G = 60$ maintaining a stable optimal state (with $\rho = 100$) within a safe path with $M_{\min }= 20$ covering continuously at least $\tau _{\min }$ percent of the path length. Additionally, we approximate the relationship between the maximum length of the stable optimal state ($\tau _{\min }'$) and the number of robots ($N_R$), for which the probability exceeds 0.975, using a non-linear regression to the exponential function $\tau _{\min }' = a \exp (-b \cdot N_R) + c$ with $a = -289.5, b = 0.1050,$ and $c = 48.86$. The residual standard error is 4.488 (on 8 degrees of freedom) after optimization over 6 iterations (with achieved convergence tolerance $8.502 \cdot 10^{-6}$). The results indicate that increasing the number of deployed robots exponentially improves the length of the stable state, up to a certain threshold ($\tau _{\min } = 50$). However, the system fails to reach a stable state for the full length of the path, suggesting that deploying redundant robots ($N_R > 32$) does not alleviate the initial stabilization process or mitigate the decline in $\rho$ at the first sharp turn (see Fig. 2B.3). Thus, the deployment of redundant robots neither enhances nor diminishes the length of the stable optimal state. In addition, Supplementary Fig. S4.1 shows that the system demonstrates fault tolerance when redundant robots are deployed.

### Shepherding success relies on successful caging, which is dependent on the robot-animal maximum velocities

**Figure 7:**
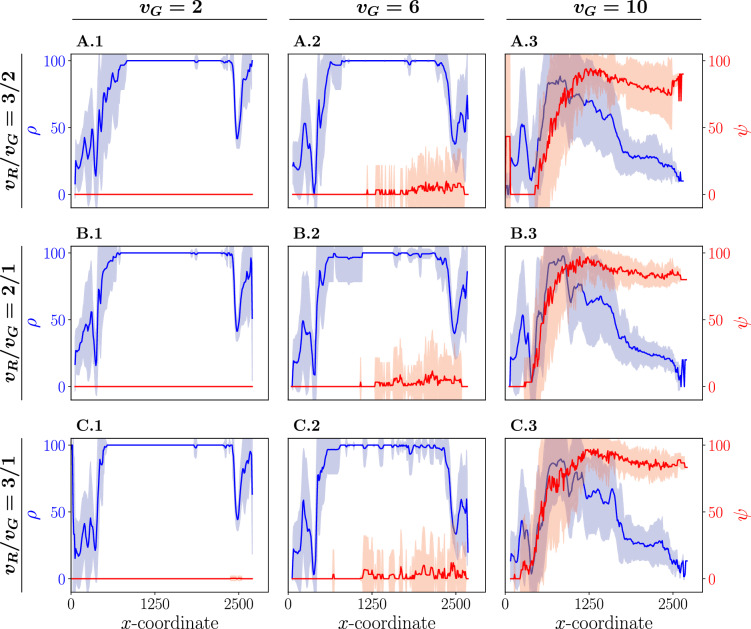
The percentage of the group within the safe path (denoted as $\rho$, colored in blue) and the percentage of the group that is not caged (denoted as $\psi$, colored in red) in function of the mean *x*-coordinate of the group. Each column considers different maximum velocities of the animals (denoted as $v_G$): $v_G = 2$ (**A-C.1**), $v_G = 6$ (**A-C.2**), $v_G = 10$ (**A-C.3**). Each row corresponds to results for different robot-animal maximum velocity ratios $\frac{v_R}{v_G} = \frac{3}{2}$ (**A.1-3**), $\frac{v_R}{v_G} = \frac{2}{1}$ (**B.1-3**), $\frac{v_R}{v_G} = \frac{3}{1}$ (**C.1-3**). The mean (solid line) and variance (shaded area) results from averaging over 30 stochastically independent simulations for each configuration, with $N_R=20$, $N_G=30$, $M_{\min }=10$, and $K = 1$.

One of the crucial parameters in this study is the maximum velocities of both the animals and the robots. In the presented algorithm, the robot must maintain its desired distance from its closest animal while remaining equidistant from its two neighboring robots. The robot predicts the movement of the fish but might have to correct prediction errors. Additionally, the robot might need to navigate from the back to the front of the group to establish and maintain the caging formation, especially in the case of malfunctioning robots. These scenarios require the robot to be able to move at a higher velocity ($v_R$) than the fish ($v_G$) in our system. In Fig. 7, the relationship between the percentage of the group inside the path ($\rho$) and the percentage that is not caged ($\psi$) is depicted as a function of the *x*-coordinate. This analysis considers varying maximum velocities for the animal, denoted as $v_G\in [2, 6, 10]$, and different robot-animal velocity ratios, expressed as $\frac{v_R}{v_G} \in [\frac{3}{2}, 2, 3]$. The results exhibit a notable similarity across all robot-animal velocity ratios, suggesting that further increases in the maximum robot velocity beyond $\frac{3}{2} v_G$ do not yield significant improvements in maintaining the group within the path.

When the animals move at a maximum velocity of 2 (A-C.1), successful caging is achieved throughout the entire trajectory, leading to effective maintenance of the group within the path. The pronounced declines in $\rho$ observed in these systems can be attributed to the specific characteristics of the path, as illustrated in Supplementary Fig. S2.1-A.1, and previously discussed in detail. As the group is able to move at a higher velocity of $v_G= 6$ (A-C.2), the caging formation becomes less consistently optimal after the group has traveled halfway along the path. Nevertheless, $\rho$ remains similar to the results obtained at $v_G= 2$. However, when the maximum animal velocity is significantly increased to $v_G= 10$ (A-C.3), $\psi$ starts to increase rapidly from the early stages of the path, reaching approximately $90\%$. This indicates that the vast majority of the group is no longer caged. Importantly, as $\psi$ increases, there is a corresponding decrease in $\rho$, signifying that the effectiveness of the caging formation is crucial to the algorithm. When this formation fails, it allows the group to venture beyond the path boundaries. Consequently, only a small percentage of the group reaches the desired goal as shown in Supplementary Fig. S1.1b, either due to random factors or because a minority of the group was steered by an incomplete caging formation. Increasing the maximum velocity of the robots does not enhance the success of caging, suggesting that other parameters may need adjustment. In the algorithm, the robots utilize two different distance thresholds to position themselves relative to a nearby animal, which are parameters of the proposed algorithm. In this paper, these thresholds are computed based on the maximum velocities of the animals, thus influencing the success of caging.

## Conclusion

Our findings offer insights into the complex dynamics of robotic agents controlling the movement of an autonomous group. We introduce a novel collective shepherding algorithm that enables a team of robots to self-organize around a group of animals (i.e. caging) and guide the group along a predefined path, contributing to safeguarding them from anthropogenic threats, including pollution and illegal exploitation. Our results have far-reaching implications for the conservation of fish and other populations, aligning with broader environmental and ecological benefits on a global scale. In the proposed algorithm, robots operate with limited knowledge about the path, relying solely on local perception and possibly communication with their direct neighbors. Our shepherding algorithm leverages the principle of robot-animal repulsion as the primary method of interaction to direct the group. As a result, the robots must collaboratively determine the distribution of forces at each of their respective positions within the cage to achieve successful steering. It is worth highlighting that our algorithm is a pioneering example of a collective shepherding approach that relies exclusively on local information to guide animals using robots. This innovative approach holds great promise for advancing the field of robotic shepherding control.

Shepherding is successfully achieved through the robots forming a caging formation around the group, and the application of repulsive forces to steer the group along the safe path, which is defined as the area between two boundary lines. The objective is for the animal group to reach the end of the path while staying within the boundaries. The effectiveness of our shepherding algorithm is profoundly influenced by a set of critical parameters. These parameters include the structural characteristics of the path, the number of robots and animals, and their maximum velocities. We observed that the robots face the greatest challenges in maintaining the group within the safe path encountering a sharp turn, especially in instances where the path narrows. Nevertheless, we noted that the severity of these disruptions tends to diminish as the space between the boundaries increases. Furthermore, hybrid systems with a 2/3 robot-animal size ratio demonstrate significant performance advantages over a 1/3 ratio. The results illustrate that deploying additional robots leads to an exponential improvement in performance. Finally, we found that when the animals move at relatively high velocity, robots do not achieve successful caging and consequently are unable to maintain the group within the safe path.

While we have demonstrated the proposed algorithm to work in simulations, there are limitations to applying it in real-life settings. First and foremost, the robots need equipment to sense the direction of the path. If only specific robots can sense this direction (e.g., when the path is illuminated, and only the front of the robot swarm can capture this information), then a communication system is also required to share this information. Additionally, the robots require sensors to measure distances and angles from nearby animals and other robots. Lastly, the robots should be capable of moving at least as fast as the maximum velocity of the fish. Apart from following the group, they need to reposition themselves from the back to the front of the group if necessary.

## Methods

### Simulation kinematics

The simulations were implemented using the Python programming language. Let $\mathscr {R}$ and $\mathscr {G}$ denote the respective sets of the robot swarm and the animal group. The state of the discrete-time system at time *t* is defined by the position $p_i(t) \in \mathbb {R}^2$ and orientation $\theta _i(t) \in [0,2\pi )$ of each individual $i \in \mathscr {R} \cup \mathscr {G}$. At every time step, the group and robots compute their new vector of motion $q_i$ respectively based on the model of collective motion or the proposed shepherding algorithm, as detailed in the following two sections. To consider physical constraints of body mass, the individual changes the motion vector with the highest priority to the average direction that is opposite to the position of all other individuals within a radius of 1 cm. To avoid collisions, the length of a motion vector is capped when it would cause the individual to be near any other individual of 1 cm distance. After computing its new motion vector, an individual first rotates with angular velocity *w* to its desired orientation $\hat{\theta }_i = \arctan (q_i)$ that is computed based on local information. The individual stops rotating once $\arctan (q_i)$ is reached, or when the time interval has passed. The individual then moves straight forward at linear velocity *v*, until the remainder of the time interval has passed or the individual has moved for a distance of $\Vert q_i\Vert$. At every time step, Gaussian noise $\sigma$ is added to the orientation of each individual.

### Collective animal motion model

We simulate the collective motion of the group as proposed by Couzin et al.^32^. Each animal *i* updates its direction of motion based on the relative positions of neighbors within a radius *z*. The neighbors are divided into three distinct subsets based on their positioning in one of the concentric zones surrounding *i*. Each zone corresponds to a different type of interaction: (i) repulsion from others inside the disk with radius $z_R$, to establish a minimum inter-individual distance, (ii) alignment of orientation with others inside the annulus with width $z_O$, to all move approximately in the same direction, and (iii) attraction to others inside the annulus with width $z_A$, to remain as one cohesive group. Following the approach of Van Havermaet et al.^4^, we extend this model by adding a zone to model the interaction between the animals and the robots : (iv) aversion from robots inside the disk with radius $z_I$, to move away from unknown mobile objects (e.g. robots) that could be predators.

Let $\mathscr {G}_i^R$, $\mathscr {G}_i^O$ and $\mathscr {G}_i^A$ denote the neighbor subsets by separating other animals of the group based on the repulsion, orientation, and attraction zones respectively. Thus, each neighbor is only assigned to one of the subsets; $\mathscr {G}_i^R \cap \mathscr {G}_i^O \cap \mathscr {G}_i^A = \emptyset$. Let $\mathscr {R}_i$ denote the subset of robots located in a radius of $z_I$ around $p_i$. Furthermore, the relative position of individual *j* from *i* is defined as $u_{ij}(t) = p_j(t) - p_i(t)$. The motion vector $q_i$ of a animal *i* is then computed as follows:

1 $$\begin{aligned} q_i(t) = - \alpha _R \sum _{j \in \mathscr {G}_i^R} \frac{u_{ij}}{\Vert u_{ij}\Vert } + \alpha _O \sum _{j \in \mathscr {G}_i^O} \frac{q_j}{\Vert q_j\Vert } + \alpha _A \sum _{j \in \mathscr {G}_i^A} \frac{u_{ij}}{\Vert u_{ij}\Vert } - \alpha _I \sum _{j \in \mathscr {R}_i} \frac{u_{ij}}{\Vert u_{ij}\Vert }, \end{aligned}$$

with weights $\alpha _R \ge 0$, $\alpha _O \ge 0$, $\alpha _A \ge 0$ and $\alpha _I \ge 0$ of repulsion, orientation, attraction, and aversive animal-robot interactions respectively.

### Safe path generation

A safe path is defined as the area between two boundaries, formed by adding equal upper and lower margins to a sequence of line segments. The sequence of segments is generated based on a given total length $L=5000$ and normal distribution of segment length $\mathscr {N}(500,\,100)$. The subsequent angles between two segments are sampled from a uniform distribution between $[0, \frac{\pi }{2}]$. As such, the path always progresses in the positive direction of the x-axis, enabling the assessment of whether the group follows the intended path direction. From the segments, we generate a discrete sequence of points $P = (X,Y)$ representing the curve at the middle of the path. The margin width *m*(*x*) is generated by a clipped mixture of *K* Gaussian distribution components. The minimum margin width $M_{\min }$ is a parameter in the experiments, while the maximum is 3 times the minimum value. The real top and bottom boundaries are defined by sequences of points $M^{t,r}$ and $M^{b,r}$ respectively. To ensure that the herd remains between the real boundaries, even under the presence of noise and the uncertainty of the progression of the path, we generate safety margin widths that are smaller than the real margin widths. Let $M^{t,s}$ and $M^{b,s}$ be the respective sequences of points representing the safety top and bottom margin boundaries. For each point *i* in *P*, we compute the approximate angle between the curve and the x-axis as $\alpha = \arctan \left( \frac{Y_{i+1} - Y{i}}{X_{i+1} - X{i}} \right)$. The top and bottom boundaries are then respectively given by the following equations:

$$\begin{aligned} M^t_i&= \left( X_i + m' \cos (\alpha + \frac{\pi }{2}), Y_i + m' \sin (\alpha + \frac{\pi }{2}) \right) \\ M^b_i&= \left( X_i + m' \cos (\alpha - \frac{\pi }{2}), Y_i + m' \sin (\alpha - \frac{\pi }{2}) \right) \end{aligned}$$

where $m' = m(X_i)$ for $M^{t,r}$ and $M^{b,r}$, and $m' = 0.6\,m(X_i)$ for $M^{t,s}$ and $M^{b,s}$. The safety margin width is thus $60\%$ of the real margin width. Note that the *M* sequences may produce loops so that the curves represented by the sequences have multiple y-values for x-values. We remove these loops in a post-processing by assigning only the minimum or maximum y-values for x-values of the top or bottom boundaries respectively.

### Robotic shepherding algorithm

The robots are tasked to steer the group, while maintaining a caging formation. The number of robots $N_R$ required to construct a caging formation depends on the size of the group $N_G$ (see Supplementary for a derivation of the lower bound on $N_R$ given $N_G$).

#### Assumption 1

The upper bound on the size of the group, $N_G$, is known a priori.

A caging formation can be constructed by the robots based on the repulsive animal-robot threshold $z_I$. When two robots are at a distance lower than $2 z_I$ of each other, they exert a combined repulsive force on the group which prevents them from intersecting the path between those robots. In our past work^4^, we proposed a decentralized algorithm for the robots to steer the group away from dynamic dangers while maintaining a caging formation. In what follows, we provide a brief description of the algorithm.

In case the robot is unable to detect any animal, the robots follow a search strategy. In this paper, we use an adaptation of the aforementioned collective animal motion model. Additionally, robots communicate their search status (i.e. if they have detected an animal) to others. Consequently, robots who are unable to directly observe any animal, will become attracted to the neighboring robot senders.

When part of the group is detected, the robot attempts to reside at a given distance of $r_c$ from the closest animal. Simultaneously, the robot moves to position itself equidistant from its two consecutive neighboring robots. During this movement, the robot may find a closer animal and will then orbit around this new animal. In other words, the robot moves along the circular perimeter of the group. To allow continuous motion along the perimeter, we hold the following assumption:

#### Assumption 2

The union of the circles, where the center points are the positions of every animal and the radii are $r_c$, is a connected set.

The robot *i* computes the desired orientation $\hat{\theta }_i$, to approach and rotate along a circular path of radius $r_c$, as proposed by Nelson et al.^33^:

2 $$\begin{aligned} \hat{\theta }_i = \gamma _i + \phi _i \left( \frac{\pi }{2} + \arctan \left( \kappa (d_i - r_i) \right) \right) \end{aligned}$$

where $\gamma _i$ is the angular position of robot *i* to the closest animal, $\phi _i \in \{-1, 1\}$ determines the direction; i.e. clockwise ($\phi _i = -1$) or counterclockwise ($\phi _i = 1$), $\kappa > 0$ influences the transition rate, $d_i$ is the distance from the robot to the animal, and $r_i$ is the desired distance. To cage, we set $r_i = r_c$ Let $\ell _{i+1}$ and $\ell _{i-1}$ be the distances of the two closest neighboring robots from opposite sides of the axis defined by $\gamma _i$. We set $\phi _i = \{-1, 1\}$ to move towards the neighbor with the highest distance. The motion vector $q_i^c = \left\langle \eta _i; \hat{\theta }_i \right\rangle$ is then combined with the predicted motion vector of the animal $q_i^g$, which is defined by the mean group velocity and the current animal orientation. The resulting motion vector $q_i$ is the summation of these two vectors.

In this paper, we adjusted the algorithm for the group to move within the boundaries of a safe path, instead of moving away from dangers. We model the information about the path in an abstract way, by using an artificial potential field. Figure 5 exemplifies an instance of a generated potential field. Let $f: \mathbb {R}^2 \rightarrow \mathbb {R}$ denote the function representing the potential at a given position of the environment. If (*x*, *y*) is located outside of the path, the potential is equal to the minimum distance from the path. Otherwise, the potential is negatively proportionate to the distance traveled along the pathway (i.e. there is a direct negative relationship between *x* and *f*(*x*, *y*)). As such, if the robot continuously moves according to the gradient $\nabla f$, it will reach the goal location with the minimum potential value. For a position $p = (x, y)$, the one-dimensional piecewise linear interpolants of *x* to $M^{t,s}$ and $M^{b,s}$ are respectively computed as $y_b$ and $y_t$. The potential function *f* is then defined as:

$$\begin{aligned} f(x,y) = {\left\{ \begin{array}{ll} f_{\min } \frac{x - x_{\min }}{x_{\max } - x_{\min }},&{} \text {if } y_b \le y \le y_t \\ \inf \{ d(p, i): i \in P \}, &{} \text {otherwise} \end{array}\right. } \end{aligned}$$

with $f_{\min }=-2000$ as the minimum potential value. The respective minimum and maximum *x*-values of *P* are denoted as $x_{\min }$ and $x_{\max }$. The function *d* computes the Euclidean distance between two points.

The gradient $\nabla f$ can also be explicitly expressed. If *p* is inside the safety path, then the gradient is equal to the derivative of *P* at $p'$, where $p'$ is the closest point of *p* to *P*. Otherwise, the gradient is equal to the direction to $p'$, i.e. $\nabla f = p' - p$. As we propose a decentralized multi-robot solution to this shepherding problem, the potential function is a way of representing locally observed information. For real-life applications, this artificial potential field can be implemented to represent various environmental stimuli, such as natural light or planned landmarks.

In order to steer the fish in the direction of $\nabla f$, we utilize the motion model of an animal as given by Eq. (1), where an animal moves in the opposite direction of a robot when their relative distance is less than $z_I$. When an agent is positioned behind all nearby animals, in the direction of $\nabla f$, then it follows Eq. (2) with $r_i = r_s$, where $r_s< z_I< r_c$. When the robot is positioned in front of the local part of the group, the robot sets $r_i = r_c$.

### Analysis

To measure the percentage of the group inside the path ($\rho$), we take for every individual position $p = (x, y)$, the one-dimensional piecewise linear interpolants of *x* to $M^{t,r}$ and $M^{b,r}$ are respectively computed as $y_b$ and $y_t$. If $y_b \le y \le y_t$, then the individual is considered to be inside the path. To measure the percentage of the group that is caged ($1 - \psi$), a caging formation can be constructed by the robotic agents based on the repulsive animal-robot threshold $z_I$. When two robots are at a distance lower than $2 z_I$ of each other, they exert a combined repulsive force on the group which prevents them from intersecting the path between those agents. In order to measure whether the robots established an appropriate caging formation, we see if a polygon can be constructed from the edges between robots where the length is shorter than $2 z_I$. Following Varava et al.^28^, we then define an animal to be caged, if and only if, the polygon is not closed and the animal is located in the interior of the polygon. To approximate the computationally intensive method of constructing a polygon from all edges (with a large number of points), the robots are first clustered. A robot is in the same cluster as its two nearest neighbors, with a relative distance lower than $2 z_I$, from opposite sides of the axis defined by the bearing of this robot from the nearest animal. If a robot does not have two neighbors, it is not in a cluster. Afterward, the convex and concave hulls are generated for each cluster. If any of these shapes are closed (i.e. each segment of the shape has a length shorter than $2 z_I$), the number of animals inside the hull is measured.

Quantifying group-path alignment involves considering the positions and directions of individual animals. Let $p_a = (x_a, y_a)$ and $q_a$ represent the position and direction of animal *a* respectively. The alignment, defined as the average alignment between each animal’s direction and the desired direction along or towards the path, is expressed as $\frac{1}{2 N_G} \sum _{a = 1}^{N_G} | \hat{q}_a + \hat{v} |$. Here, $\hat{q}_a$ is the unit direction vector, and $\hat{v}$ is the unit vector of the gradient $\nabla f(x_a, y_a)$ based on the potential field function *f* (refer to the previous subsection). To assess the compression of the animal group, a convex hull is constructed for all animal positions. From this convex hull, the minimum bounding rectangle (also known as the bounding box) is generated. The compression is then determined by the ratio of the smaller (width) to the larger (length) dimensions of this rectangle.

### Simulation parameters

The simulation parameters are based on our previous work^4^ and are shown in Supplementary Table S2.1. The parameters of the animals are inspired by live Trinidadian guppies, who live in shallow waters, which applies to our problem setting of a two-dimensional environment. The parameters of the robots are based on the work of Landgraf et al.^34^ designing fish-like robots.

### Supplementary Information

Supplementary Information.

## Data Availability

The datasets generated and/or analysed during the current study are available in the Open Science Framework repository; https://osf.io/5xr3t. All code to produce said data is available at: https://github.com/stefvh/path_shepherding.
