# Supplementary material for: Reactive shepherding along a dynamic path
Source: Sci Rep. 2024 Jun 28;14:14915. doi: 10.1038/s41598-024-65894-5 (PMC11213918; doi:10.1038/s41598-024-65894-5)
Supplement: Supplementary file 1 — Supplementary Information. [file 41598_2024_65894_MOESM1_ESM.pdf]

Supplementary Materials
Reactive shepherding along a dynamic path

Stef Van Havermaet, Yara Khaluf, and Pieter Simoens

1 Percentage of the group reaching the desired goal

Figure S1.1 shows the percentages of the group reaching the desired goal for varying parameter configurations. A comparison of Figure 2 and Figure S1.1a indicates that maintaining the group on the safe path near the end ensures reaching the desired goal. From Figure S1.1a, we observe that 20 robots cannot guarantee that all 60 animals reach the desired goal under the considered configuration parameters. In Figure S1.1b, we observe that the percentage of animals reaching the desired goal decreases with increasing maximum velocity v_G.

2 Performance for $K = 1$

Supplementary Figure S2.1 displays the percentage of the group within the safe path (ρ) and the percentage of the group not caged (ψ), as a function of the mean x-coordinate of the group. Each column showcases outcomes for distinct system sizes (i.e. the number of robots N_R, and the number of animals in group N_G), while each row varies in the minimum margin width $M_{\min}$ (A: 10, B: 20, C: 50) used to generate the margins. Although the resulting paths consist of the same series of line segments, they consequently differ in margin widths on both sides of these segments. A visual representation of each path is provided as an inset in the first column of the corresponding row (A-C.1).

For all considered values of $M_{\min}$, the systems with a size ratio of 2/3 (i.e. sizes 20/30 and 40/60) consistently reach a stable optimal state, wherein the entire group remains within the safe path ($\rho = 100$) continuously for a substantial length of the path. Systems with a 1/3 size ratio only reach a stable state when the minimum margin is at its largest (C.2). To attain this stable optimal state, these systems require some initial distance, which diminishes as the minimum margin $M_{\min}$ increases. While observing the initial stabilization process, we note a significant disruption for most configurations at a specific point along the path where

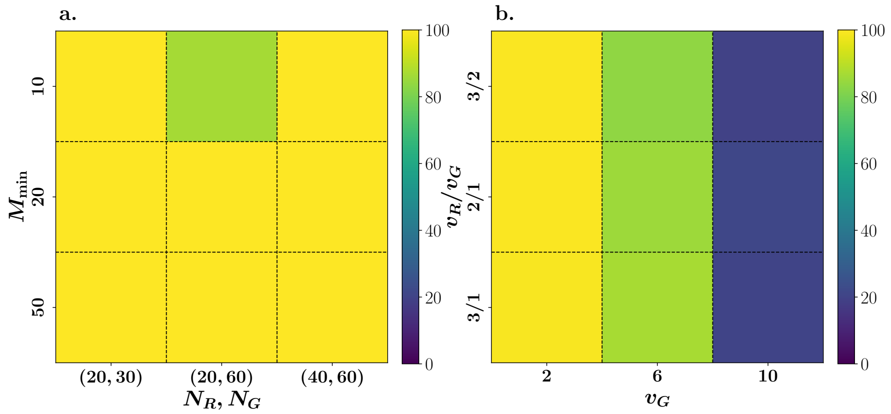

Figure 1.1: Percentages of the group reaching the desired goal under varying configuration parameters. **(a)** and **(b)** respectively correspond to the simulations presented in the manuscript in Figure 2 and 7. Results are averages over 30 stochastically independent simulations. Configuration of **(a)** includes $v_R = 4$, $v_G = 2$ and $K = 3$. Configuration of **(b)** includes $N_R = 20$, $N_G = 30$, $M_{\text{min}} = 10$ and $K = 1$.

the margin width is minimal and a sharp turn occurs. Supplementary Figure S2.1 indicates this position by an open orange circle. Despite the sudden decline in ρ at this point, the group remains fully caged ($\psi = 0$), enabling these systems to recover and eventually reach a stable optimal state. However, this state is eventually disrupted by another point along the path, marked by an open red triangle in Supplementary Figure S2.1. Again, this location is characterized by a minimal margin width and a sharp turn. We observe that these disruptions were mitigated to a lesser extent with an increase in the minimum margin width. Indeed, for $M_{\text{min}} = 50$, the stable optimal state of a system with size 20/30 remains unaffected by the aforementioned scenarios.

Finally, when comparing the initial distance to stabilize with the group contained within the safe path between $K = 1$ (Supplementary Figure S2.1) and $K = 3$ (Main Figure 2), we notice that the path with margin width generated by $K = 3$ Gaussian distributions exhibits a significantly earlier stabilization point. This can be attributed to the distinctive characteristics of the single Gaussian component ($K = 1$), which tends to produce a longer narrow segment at the start and end of the path, given that the total length of the path remains constant for both $K = 1$ and $K = 3$. As a consequence, the robots struggle with aligning the group within the path at the beginning, causing a delay in achieving the desired level of stability. For this reason, the declines for $K = 3$ at the first and last turn of the path, respectively indicated by an open orange circle and red triangle, are less substantial in comparison to $K = 1$.

3 Worst-case lower bound on the number of robots

We estimate the minimum number of robots N_R needed to cage a group of size N_G in the worst-case scenario, where caging is defined as follows:

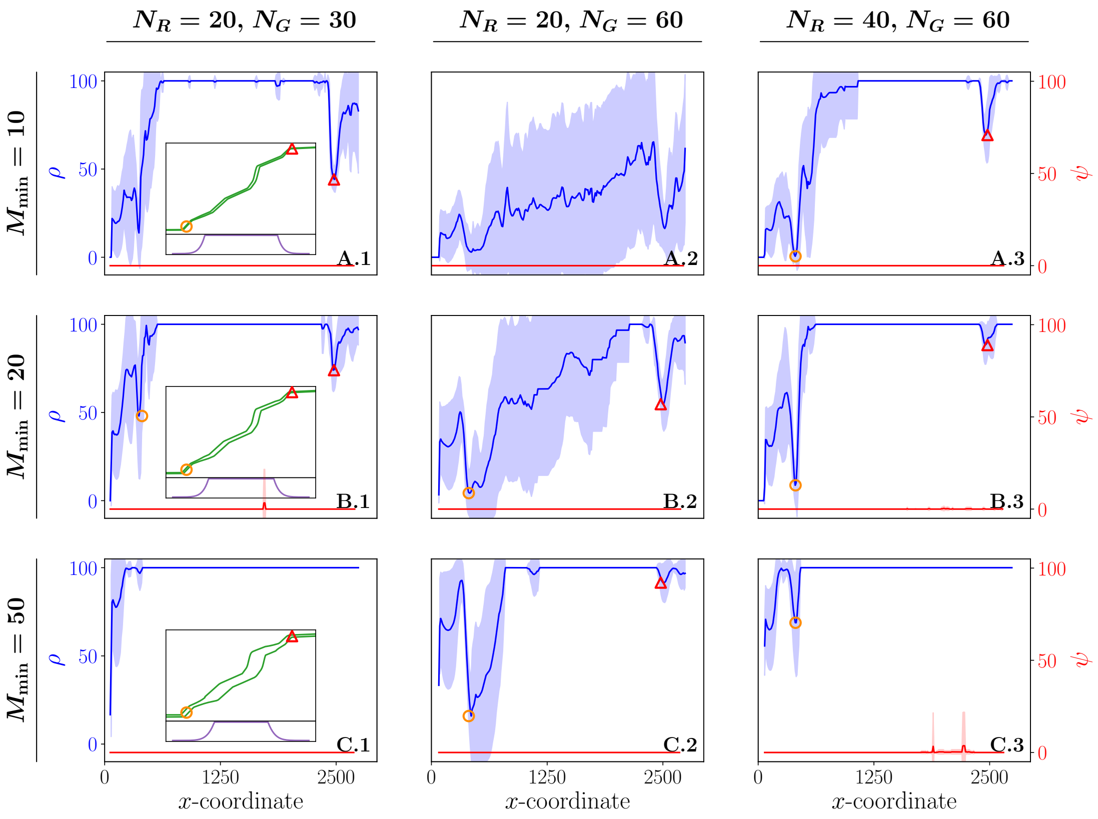

Figure 2.1: The percentage of the group within the safe path (denoted as ρ, colored in blue) and the percentage of the group that is not caged (denoted as ψ, colored in red) in the function of the mean x-coordinate of the group. Each row corresponds to simulation results for different values of the minimum margin width $M_{\min}$ of the path: 10 (**A.1-3**), 20 (**B.1-3**), 50 (**C.1-3**). Each column considers different sizes of the system (i.e. the number of robots N_R and the size of the group N_G): $N_R = 20, N_G = 30$ (**A-C.1**), $N_R = 20, N_G = 60$ (**A-C.2**), $N_R = 40, N_G = 60$ (**A-C.3**). The first column shows the corresponding path of its row as an inset. Significant declines in performance are marked by different colors and symbols: an open red triangle, and an open orange circle. The location on the path where these declines happen is correspondingly marked in the insets. The mean (solid line) and variance (shaded area) of ρ and ψ results from averaging over 30 stochastically independent simulations for each configuration, with $K = 1$.

Definition 1. *In a caging formation, each robot maintains a distance of r from their respective closest animal, while positioning themselves at equal distance from their two neighboring robots. These two neighbors are positioned on opposite sides of the axis defined as the line intersecting the positions of the focal robot and their closest animal. The distance between consecutive robots should be at most* $2r$.

From a geometrical perspective, each animal can be considered to construct a circle with its position as the center and a radius of r. Assuming the union of these N_G circles forms a connected set, the animal group can then be represented by a single shape.

Assumption 1. *The union of the circles, where the center points are the positions of every animal and the radii are equal to* r*, is a connected set.*

Under these conditions, positioning N_R robots in a caging formation surrounding the group corresponds to uniformly distributing N_R points along the perimeter P of this shape. We then derive an estimate of the absolute minimum N_R points needed for a spatial arrangement of the group that maximizes P. In such an arrangement, the circles touch (Assumption 1) but do not overlap more than necessary. An example of this is N_G circles in a linear arrangement, where the distance between consecutive circle center points is $2r-\varepsilon$, with ε being a small value that ensures minimal overlap. We will derive the following equations based on this spatial arrangement.

The perimeter P can then be approximated as the sum of all individual circles minus the parts hidden by the overlaps. To calculate the overlap, we find the intersection points (x_e, y_e) of consecutive circles, which can be approximated for small ε as follows:

$$x_e \approx r - \frac{\varepsilon}{2}$$
$$y_e \approx \pm\sqrt{r\varepsilon}$$

The length of the shortest arc ℓ_e between these two points can then be computed as $\ell_e = r\theta_e$ where θ_e is the angle subtended by the chord at the center of one circle, given by:

$$\theta_e = 2\cos^{-1}\left(1 - \frac{\varepsilon}{2r}\right)$$
$$\theta_e \approx 2\sqrt{\frac{\varepsilon}{r}}$$

Thus, the length that should be removed from the perimeter of each circle for each overlap is approximately $\ell_e \approx 2\sqrt{r\varepsilon}$. The first and last circles will have one overlap, while each other circle has two distinct overlaps. Therefore, the perimeter P of the shape defined by N_G circles can be approximated as:

$$P \approx 2\pi r N_G - 4(N_G - 1)\sqrt{r\varepsilon}.$$

Next, we estimate how many points N_R are required to be equidistantly distributed on this perimeter with maximum distance $2r$ between them. For the robots, we lower the maximum distance to $z = 2(r - e)$ with $e \ll r$, to account for the physical size of the robot and the their stochastic actuators and sensors. The perimeter P is an accumulation of (curved) arc

lengths of every circle. In order to measure a ratio between P and z, we must translate z to arc length. Let z be a chord length, then the central angle θ_z subtended by the chord of length z is approximated by Taylor series expansion as

$$\begin{aligned}\theta_z &= 2\sin^{-1}\left(1-\frac{e}{r}\right)\\ &\approx \pi - 2\sqrt{\frac{2e}{r}}.\end{aligned}$$

The arc length corresponding to this angle is then $\ell_z = \pi r - 2\sqrt{2re}$. The lower bound on N_R is given by $\frac{P}{\ell_z}$. Thus, to cage N_G animals behaving under Assumption 1, at least N_R robots are required, as given by the following inequality:

$$N_R \geq \frac{2\pi r N_G - 4(N_G-1)\sqrt{r\varepsilon}}{\pi r - 2\sqrt{2re}}. \tag{3.1}$$

We note that the lower bound (3.1) of N_R is derived when the group is exactly positioned to maximize the group perimeter. However, in our simulations, we observe that the spatial arrangement of the group gradually conforms to the shape of the path, eventually becoming highly elongated (see Figure 5b in the main manuscript, where $M_{\min} = 10$). This spatial arrangement resembles the one used to derive the lower bound. Nevertheless, our simulations indicate that the robots can maintain the cage with fewer robots than the calculated lower bound when ε is small (i.e. the group is not tightly connected). This suggests that the dynamics of the animal motion model and the robot control laws used in our simulations result in a more tightly connected group.

4 Fault tolerance

Supplementary Figure S4.1 presents the percentage of the group within the safe path (ρ), the percentage of the group not caged (ψ), and the number of active robots (N_R^{act}) as functions of time t. At each time step t_F, one robot is uniformly randomly selected to malfunction from the set of robots positioned at l_F. To ensure a minimally functioning system, malfunctions do not occur when only 3 are currently functioning ($N_R^{\text{act}} = 3$), or when there is no active robot positioned at l_F. The system's performance of 20 robots and 30 animals is observed through two variables: (i) Δt_F, representing the time between consecutive malfunctions, and (ii) l_F, the coordinate relative to the group's frame of reference, with $l_F \in [BACK, FRONT, LEFT]$. The direction $RIGHT$ is omitted due to symmetry with $LEFT$.

With a fault interval of $\Delta t_F = 200$ (A.1-3), the robots consistently maintain a cage formation, ensuring all animals of the group remain inside the cage ($\psi = 0$) even as robots periodically malfunction, until less than a critical number of robots remain operational. However, upon comparing failures across different group-centric coordinates (l_F), it becomes evident that this ability to keep the entire group within the cage diminishes much faster when robots malfunction in front of the group compared to malfunctions occurring on the left side or at the back. As the number of operational robots decreases, a certain percentage of animals is left uncaged each time a robot malfunctions. Nevertheless, the robots continuously manage

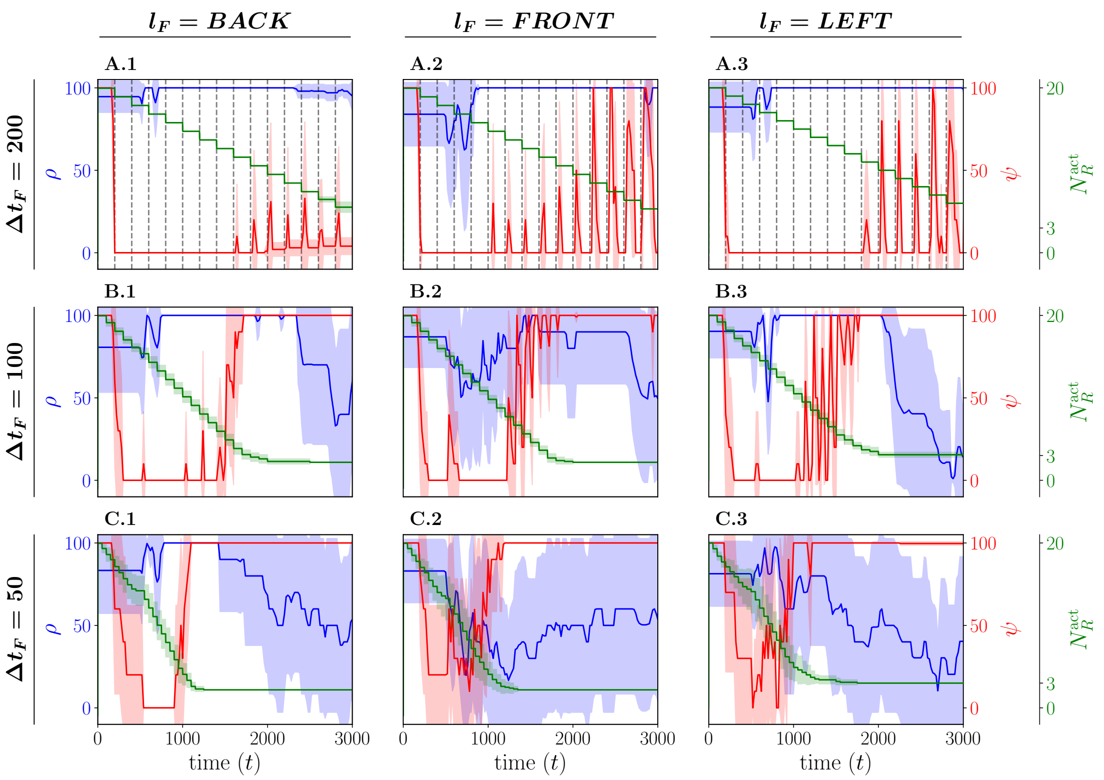

Figure 4.1: The percentage of the group within the safe path (denoted as ρ, colored in blue), the percentage of the group that is not caged (denoted as ψ, colored in red), and the number of active robots (denoted as N_R^{act}, colored in green) in the function of time t. At each time step t_F, a robot is randomly selected to malfunction from the set of robots positioned at l_F. Each row corresponds to simulation results for different values of the fault interval Δt_F which determines the duration between consecutive faults: 200 (**A.1-3**), 100 (**B.1-3**), 50 (**C.1-3**). Each column considers different coordinates relative to the group's frame of reference l_F: $BACK$ (**A-C.1**), $FRONT$ (**A-C.2**), $LEFT$ (**A-C.3**). The time steps t_F at which a robot malfunctions are indicated by vertical dashed lines. While they are displayed for configurations in the first row, they are omitted in subsequent rows for better visibility. The mean (solid line) and variance (shaded area) of ρ and ψ results from averaging over 10 stochastically independent simulations for each configuration, with $N_R = 20, N_G = 30, M_{\min} = 50$, and $K = 1$.

to re-establish their caging formation within the $\Delta t_F = 200$ duration before another robot becomes inactive. This recovery period appears to lengthen as the number of functioning robots decreases, and correspondingly, the percentage of the group that remains uncaged during recovery seems to increase as the number of active robots decreases. It is noteworthy that despite temporary difficulties in keeping the group caged, the group consistently remains fully within the safe path ($\rho = 100$). This resilience is facilitated by the sufficiently wide margin width ($M_{\min} = 50$), which allows for temporary issues without compromising the group's safety within the designated path. s Δt_F is reduced to 100 (B.1-3) and 50 (C.1-3), it becomes apparent that the robots reach a point where they are unable to restore the cage formation when the number of functioning robots is approximately less than 10. Consequently, the group remains entirely uncaged, allowing the animals to move freely. This lack of containment may lead to the group deviating from the safe path.

5 Varying robot velocity for high-speed animals in straight-line paths

In the manuscript, we have shown that the robots significantly fail to cage the vast majority of the group when the maximum animal velocity is set to $v_G = 10$, independent of the maximum robot velocity v_R. Consequently, the group moves freely and a significant part of the group leaves the safe path. In order to investigate whether the path characteristics play a role in the ability to maintain a caging formation, we have run additional simulations where the path has no turns (i.e. the path represents a horizontal line from the starting position to the desired goal). Supplementary Figure S5.1 demonstrates how the percentage of the group within the safe path (ρ) and the percentage of the group not caged (ψ) as functions of the x-coordinate of the group, for various values of maximum robot velocity v_R. In comparison to the results presented in the manuscript for dynamic paths, it seems that the significantly low performance for high-speed animals is not related to path characteristics. We therefore hypothesize that the failure in caging high-speed animals may be due to the prediction error of the animal direction that is used in the robotic caging algorithm. These directional prediction errors cause larger positional errors when velocity of the animals is increased. A first priority of future work is to address this issue.

6 Simulation parameters

Supplementary Table S6.1 provides a comprehensive overview of the simulation parameters governing the dynamics of the robotic and animal entities within the two-dimensional environment. The parameters are categorized based on their association with animals, robots, and the path, each contributing to the simulation's realism and relevance. The parameters of the animal group are inspired by live Trinidadian guppies, who live in shallow waters, which applies to our problem setting of a two-dimensional environment. The robot parameters are derived from two-wheeled motion robots.

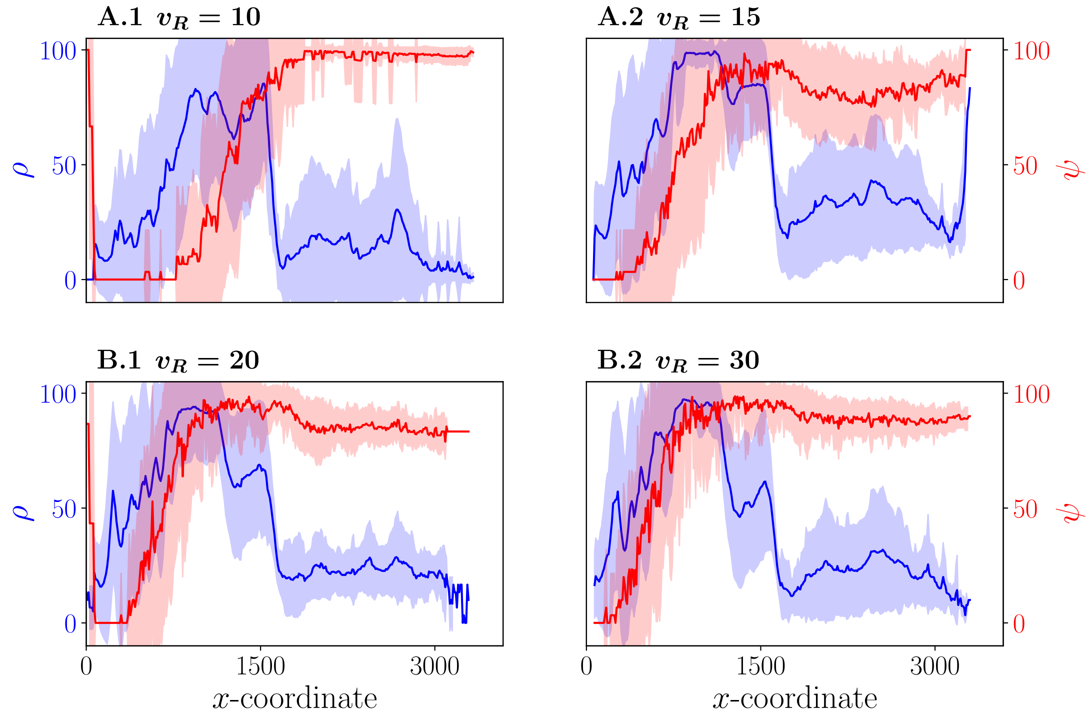

Figure 5.1: The percentage of the group within the safe path (denoted as ρ, colored in blue), and the percentage of the group that is not caged (denoted as ψ, colored in red), in the function of the x-coordinate of the group. The robots are tasked with shepherding a high-speed animal group along a safe path without turns. The sub figures show results of various maximum velocities of the robot v_R. The mean (solid line) and variance (shaded area) of ρ and ψ results from averaging over 10 stochastically independent simulations for each configuration, with $N_R = 20$, $N_G = 30$, $M_{\min} = 10$, and $v_G = 10$.

symbol	parameter	value	association
N_G	size of the group	$\{30, 60\}$	animal
N_R	size of the robot swarm	$[20, ..., 40, ..., 60]$	robot
M_{min}	minimum margin width	$\{10, 20, 50\}$	path
K	number of Gaussian distribution components	$\{1, 3\}$	path
v_G & v_R	max. linear velocity	2 & 4 $\frac{\text{cm}}{\text{s}}$	animal & robot
w_G & w_R	max. angular velocity	π & π $\frac{\text{rad}}{\text{s}}$	animal &robot
σ_G & σ_R	Gaussian orientation noise	0.05 & 0.05 rad	animal & robot
z_R	radius of repulsion zone	1 cm	animal
z_O	radius of orientation zone	50 cm	animal
z_A	radius of attraction zone	30 cm	animal
z_I	radius of aversive zone	40 cm	animal
α_R	weight of repulsion interaction	100	animal
α_O	weight of orientation interaction	50	animal
α_A	weight of attraction interaction	1	animal
α_I	weight of aversion interaction	100	animal
d_d	detection radius	50 cm	robot
κ	transition rate	0.2	robot
r	relative distance robot-animal to observe	$z_I + v_G$ cm	robot
R	relative distance robot-animal to steer	$z_I - v_G$ cm	robot

Table 6.1: Simulation parameters. For each parameter, the table lists: (i) the symbol used for reference in the main manuscript (or refers to Van Havermaet et al. [1] if not present in this manuscript), (ii) the name of the parameter, (iii) the value(s) employed in the experiments, and (iv) the association indicating whether it relates to the animal, robot, or path.

References

[1] Stef Van Havermaet et al. “Steering herds away from dangers in dynamic environments”. In: *Royal Society Open Science* 10.5 (2023), p. 230015.
